# Autoimmune-associated epilepsy: definitions and diagnosis – a narrative review

**DOI:** 10.1055/s-0046-1827055

**Published:** 2026-08-31

**Authors:** Tomásia Oliveira de Holanda Monteiro Frezatti, Vanessa Daccach Marques, Américo Ceiki Sakamoto

**Affiliations:** 1 Universidade de São Paulo, Faculdade de Medicina de Ribeirão Preto, Departamento de Neurociências e Ciências do Comportamento, Ribeirão Preto SP, Brazil.

**Keywords:** Epilepsy, Autoimmune Diseases, Antibodies, Neuronal, Biomarkers

## Abstract

Autoimmune-associated epilepsy (AAE) is an increasingly-recognized condition in which seizures result from immune-mediated mechanisms, such as Rasmussen's encephalitis and antibody-associated epilepsies. Its timely identification may enable more efficacious therapy and improve outcomes. The current paper aims to provide a clinically-oriented review of AAE, focusing on clinical identification, use of diagnostic scoring systems, and immunological testing strategies. Antibody-associated epilepsy encompasses a broad spectrum of presentations, from postencephalitic epilepsy to isolated drug-resistant temporal-lobe epilepsy. Neuronal-surface autoantibodies (such as anti-N-methyl-D-aspartate receptor (anti-NMDAR), anti- leucine-rich glioma-inactivated protein 1 (anti-LGI1), anti-CASPR2) and intracellular antibodies (such as anti-GAD65) are variably associated with pathogenesis and chronicity. Clinical scoring systems (such as Antibody Prevalence in Epilepsy and Encephalopathy - APE2, Antibody Contributing to Focal Epilepsy Signs and Symptoms - ACES, ntibody Prevalence in Epilepsy before Surgery - APES, Antibody in Drug-Resistant Temporal Lobe Epilepsy - ARTE and ‘Obvious’ Indications for Neural Antibody Testing in Epilepsy or Seizures - ONES) are helpful in selecting patients for autoantibody testing. A comprehensive workup includes magnetic resonance imaging (MRI) scans, prolonged electroencephalographic (EEG) monitoring, cerebrospinal fluid (CSF) analysis, and combined serum/CSF antibody panels. Testing methodology and antibody type must be carefully interpreted in the light of clinical context. In low-resource settings, cost-effective testing strategies and clinical-screening tools are crucial to optimize the diagnostic yield. Early clinical suspicion, guided use of diagnostic scores, and appropriate immunological investigation are central to manage AAE. Recognizing AAE as a distinct diagnostic category is essential to improve care and guide immunotherapeutic decisions.

## INTRODUCTION

Epilepsy is a neurological disorder that affects ∼ 0.5 to 1% of the global population. 1 2 Although defining the underlying etiology is crucial for management and prognosis, nearly 1/3 of epilepsy cases remain of unknown cause. Among these, a subset of patients may have an immune-mediated pathogenesis and may benefit from immunotherapy in addition to conventional antiseizure medications (ASMs). 3 4

The concept of *autoimmune epilepsy* was first introduced in 2002 at the International Congress of Autoimmunity, initially focusing on autoimmune encephalitis and Rasmussen's encephalitis. 2 Subsequent studies 4 5 reported an increased incidence of seizures in individuals with systemic autoimmune conditions such as systemic lupus erythematosus and type-1 diabetes mellitus, raising hypotheses of shared immunopathological mechanisms and leading to the progressive expansion of the autoimmune epilepsy spectrum.

In 2016, Britton 6 proposed a classification under the umbrella term *autoimmune epilepsy syndromes* , which included 4 categories: primary autoimmune epilepsies; epilepsies responsive to immunotherapy; autoimmune disorders associated with epilepsy; and antibody-associated epilepsy syndromes. This framework emphasized the heterogeneity of autoimmune-related epileptic conditions, encompassing persistent seizure disorders such as Rasmussen's encephalitis and transient syndromes such as limbic encephalitis.

In 2017, the International League Against Epilepsy 7 (ILAE) updated its classification to incorporate 6 etiological categories, including immune etiology. Immune epilepsy was defined as epilepsy secondary to an autoimmune disorder in which seizures constitute the primary clinical manifestation. However, examples cited, such as anti-N-methyl-D-aspartate receptor (anti-NMDAR) and anti-leucine-rich glioma-inactivated protein 1 (anti-LGI1) encephalitis often involve only acute symptomatic seizures during the inflammatory phase, rather than chronic epilepsy. 8

To address this distinction, in 2020, the ILAE Autoimmunity and Inflammation Task Force published a position paper 9 formally differentiating between “acute symptomatic seizures secondary to autoimmune encephalitis” and “autoimmune-associated epilepsy” (AAE). The authors 9 recommended discontinuing the term *autoimmune epilepsy* , highlighting that non-immune mechanisms (such as structural sequelae) may contribute to seizure generation, and that the term could misleadingly suggest persistent inflammation requiring ongoing immunotherapy.

Despite the increasing recognition of immune mechanisms in epilepsy, substantial heterogeneity persists regarding the diagnostic thresholds, the interpretation of antibody positivity, and the therapeutic decision-making. Overestimation of antibody relevance, variability in testing methodologies, and conflation of acute encephalitic seizures with chronic epilepsy have contributed to conceptual ambiguity. In this context, a structured, probability-based clinical framework becomes essential to avoid underdiagnosis and inappropriate immunosuppression.

In the current narrative review, we aim to summarize the available evidence and to critically synthesize the current diagnostic tools, contextualize antibody-testing strategies, and propose a pragmatic clinical approach to AAE, with particular attention to real-world constraints in low- and middle-income settings. Table 1 summarizes the main concepts discussed.

**Table 1:** Main concepts related to autoimmune-associated epilepsy

Concept	Definition
Autoimmune-associated epilepsy	Enduring predisposition to seizures secondary to an immune-mediated brain disease, such as Rasmussen's encephalitis and antibody-associated epilepsy
Acute symptomatic seizures secondary to autoimmune encephalitis	Seizures in the context of autoimmune encephalitis at initial or relapsing presentations
Antibody-associated epilepsy	Autoimmune-associated epilepsy with positivity to autoantibodies against neuronal (intracellular or surface) antigens

## METHODS

The present narrative review was conducted through a structured literature search in the PubMed/MEDLINE and Google Scholar databases. The search included articles published up to April 30, 2025, using the following keywords in the title and/or abstract: *autoimmune epilepsy* , *autoimmune seizures* , *autoimmune-associated epilepsy* , and *antibody and epilepsy* . Original articles, systematic reviews, narrative reviews, and relevant book chapters published in English were considered eligible. The reference lists of the selected articles were manually screened to identify additional pertinent studies. The search yielded ∼ 3,500 records. Titles and abstracts were screened for relevance to the scope of the review, focusing on clinical definition, pathophysiology, diagnostic criteria, biomarkers, and treatment of AAE. Articles primarily addressing classic autoimmune encephalitis without a specific discussion on epilepsy as a chronic or predominant manifestation were excluded, unless they were directly relevant to conceptual or therapeutic aspects. The final reference list was developed based on methodological quality, clinical relevance, and contribution to the understanding of AAE.

## IMMUNOPATHOGENESIS OF AUTOIMMUNE-ASSOCIATED EPILEPSY

The pathogenesis and ictogenesis of AAE may involve cytotoxic mechanisms (leading to neuronal injury) and the direct effects of autoantibodies targeting neuronal-surface proteins, thereby promoting neuronal hyperexcitability. 10

In Rasmussen's encephalitis, for example, the immune response is primarily mediated by cytotoxic CD8 +  T cells, natural killer (NK) cells, and activated microglia, resulting in pronounced neuroinflammation and neuronal necrosis. In contrast, antibody-associated epilepsies involve B and T lymphocytes, which contribute to neuronal dysfunction, inflammatory cascades, and structural alterations. These processes may manifest as acute symptomatic seizures and, in some cases, progress to AAE. 4

The underlying immunopathological mechanisms vary depending on the antigenic target. When the autoantigen is located intracellularly, the immune response is predominantly mediated by T cells. This raises an ongoing debate regarding the direct pathogenicity of such antibodies, as they may function primarily as disease biomarkers. Notably, patients with antibodies against intracellular antigens exhibit a higher risk of evolving into chronic epilepsy compared with those with antibodies targeting neuronal-surface antigens. 4 10 11

Neuronal surface antibodies are considered to have a more direct pathogenic role. These antibodies may induce neuroinflammation via antigenic modulation, internalization of surface receptors, and/or activation of immune effector pathways, including complement cascade recruitment and immune cell infiltration. 12

Chronic AAE arises when sustained immune-mediated processes induce lasting alterations in neuronal networks, creating a persistent susceptibility to unprovoked seizures. The antibody-mediated dysfunction is amplified by complement activation, microglial reactivity, and cytotoxic T-cell infiltration, all together sustaining neuroinflammation and neuronal injury. 8

## AUTOANTIBODIES AND EPILEPSY

Although the term *autoimmune epilepsy* was first introduced in 2002, the hypothesis that certain forms of epilepsy may have an immune-mediated etiology dates to more than a century. This concept was revisited in the 1960s and 1970s following experimental studies demonstrating that the infusion of antibodies into the ventricles and brains of cats and monkeys induced hyperexcitability and epileptiform activity. In the 1980s and 1990s, the discovery of immune responses directed against specific antigens of the central nervous system (CNS) in paraneoplastic syndromes provided compelling evidence 8 that autoimmunity targeting neuronal proteins could result in severe forms of encephalitis frequently associated with seizures.

Subsequent findings later reinforced this concept. Rabbits immunized with the glutamate receptor 3 (GluR3) subunit of the α-amino-3-hydroxy-5-meth-yl-4-isoxazolepropionic acid (AMPA) receptor developed seizures and histopathological features resembling those of Rasmussen's encephalitis. 8 Parallel to the identification of antibodies in patients with autoimmune encephalitis, case reports and series 13 14 emerged, describing individuals with isolated seizures and positive autoantibodies, as well as patients with chronic epilepsy of unknown etiology who tested positive for neuronal antibodies in serum and/or cerebrospinal fluid (CSF).

Evidence 15 supporting the pathogenicity of these antibodies includes the temporal correlation between clinical improvement and reduction in antibody titers, their detection in the CSF, and the reproduction of key disease features in both in-vitro and in-vivo models using patient-derived antibodies. These observations have led to the recognition of antibody-associated epilepsy as a well-defined entity within the broader spectrum of AAEs.

**Figure 1:**
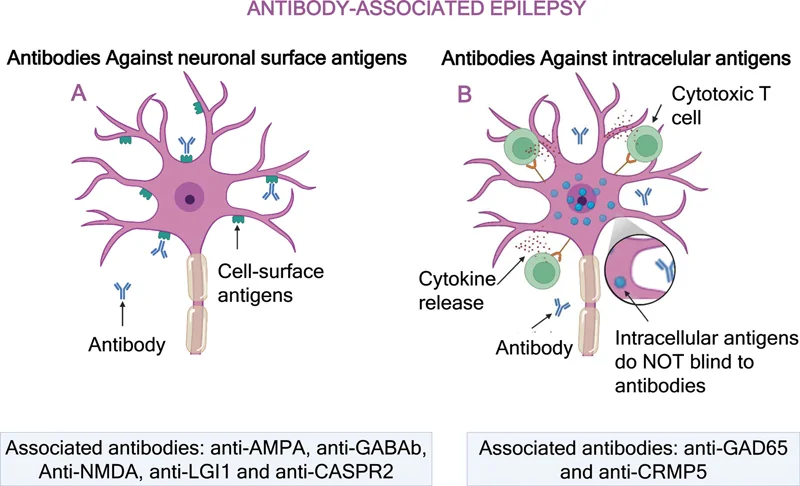
Note: Figure created with the BioRender (Science Suite Inc.) tool. Main autoantibodies related to autoimmune-associated epilepsy. ( **A** ) In epilepsy associated with antibodies targeting cell-surface antigens, the antibodies have access to their targets and can alter the structure and function of the antigen. ( **B** ) In epilepsy associated with antibodies against intracellular epitopes, the antibodies cannot reach the intracellular antigens, and cytotoxic T-cell mechanisms are mainly involved, leading to neuronal loss.

Neuronal autoantibodies are commonly classified based on whether they target extracellular (neuronal surface) or intracellular antigens. The main antibodies associated with epilepsy are illustrated in Figure 1 . Key surface antigens include LGI1, contactin-associated protein-like 2 (CASPR2), NMDAR, and γ-aminobutyric acid receptors (GABAR), while glutamic acid decarboxylase (GAD) is the most frequently-described intracellular target. 15 Experimental studies involving these targets have shown that antibody modulation may reduce epileptogenicity. 16

Some antibodies, such as voltage-gated potassium channel (VGKC) antibodies without LGI1 or CASPR2 specificity, or low-titer anti-GAD65 antibodies have so far unclear clinical relevance. This contributed to inconsistencies in published data, particularly in prevalence studies, and methodological variability. 17

Although neuronal autoantibodies are most associated with acute symptomatic seizures in autoimmune encephalitis, they may also be found in patients with epilepsy lacking overt psychiatric or cognitive symptoms. 18 Therefore, AAE may present in two distinct clinical contexts: following an unrecognized acute encephalitic phase. in which immunotherapy was not administered promptly, or as isolated epilepsy, without preceding encephalitic symptoms. In the latter scenario, anti-GAD65 is the most frequently-identified autoantibody. 14

While patients with high-titer anti-GAD65 antibodies may present with a range of syndromes, including limbic encephalitis, cerebellar ataxia, and stiff-person syndrome, up to 57% of the patients with temporal-lobe epilepsy (TLE) and anti-GAD65 antibodies may exhibit isolated epilepsy. This suggests that anti-GAD65 is likely an underdiagnosed cause of drug-resistant TLE. 19

However, caution is warranted when interpreting low-titer anti-GAD65 antibodies. Approximately 85% of the patients with newly-diagnosed type-1 diabetes harbor GAD65 antibodies, often at low titers. Neurological manifestations, including epilepsy, are typically associated with high antibody titers, and paired serum–CSF analysis is particularly helpful in suspected cases. 5

Regarding clinical semiology, certain seizure types may well raise the suspicion of immune etiology. These include perisylvian semiology, 18 reflex musicogenic seizures, 20 and seizures with autonomic features, such as piloerection. 21 Importantly, seizure frequency in AAE is often underestimated, as patients and families may fail to recognize cognitive, emotional, or autonomic events, and they may not report seizures with impaired awareness or those occurring during sleep. For instance, a study 22 using prolonged video-electroencephalography (VEEG) monitoring in patients with LGI1 and CASPR2 antibody-associated epilepsy found that 20% of the individuals who had self-reported as seizure-free had electrographic seizures.

Despite growing interest in the prevalence and treatment of epilepsy in individuals with neuronal autoantibodies, several limitations persist. Many studies fail to rigorously exclude patients with autoimmune encephalitis, and most rely on serum-only testing, often without confirmatory CSF analysis. Additionally, substantial methodological heterogeneity limits comparisons across studies. 17

It is essential to distinguish antibodies predominantly associated with acute autoimmune encephalitis from those more robustly linked to chronic AAE. In autoimmune encephalitis, the seizures usually occur as acute symptomatic events accompanied by cognitive dysfunction, psychiatric symptoms, movement disorders, or autonomic instability. In contrast, chronic AAE is characterized by recurrent unprovoked seizures that persist beyond the inflammatory phase, often without overt encephalitic features. 9

The strength of the evidence supporting chronic epilepsy varies substantially across antibodies. High-titer anti-GAD65 and anti-LGI1 antibodies show more consistent associations with persistent seizure disorders, particularly TLE, in observational cohorts. Conversely, anti-NMDAR antibodies, although frequently associated with seizures during acute encephalitis, rarely lead to chronic epilepsy once the inflammatory process resolves. 9 In a prospective, multicenter cohort of 582 adults with focal epilepsy of unknown etiology and no previous clinical suspicion of acute encephalitis, 23 neuronal autoantibodies were identified in 20 patients (3.4%), including anti-LGI1 ( *n* = 3), anti-CASPR2 ( *n* = 3), anti-NMDAR ( *n* = 1), and high-titer anti-GAD65 antibodies ( *n* = 13).

In clinical practice, antibody panels for suspected AAE should be as comprehensive as possible. The inclusion of antibodies against the GABAB receptor, NMDA receptor, LGI1, CASPR2, GAD65, and AMPA receptor is suggested, as these are the most clinically-relevant in this context, and testing should be performed in both serum and CSF.

## RECOGNIZING ANTIBODY-ASSOCIATED EPILEPSY IN CLINICAL PRACTICE

Identifying patients with an underlying antibody-associated etiology remains a significant challenge in clinical practice. Broad neuronal antibody testing in individuals with low pretest probability is not recommended, as it may lead to the detection of antibodies with uncertain clinical significance, inappropriate initiation of immunotherapy, and unnecessary increase in healthcare costs. Thus, antibody testing should be reserved for individuals with clinical features suggestive of AAE and a reasonable likelihood of benefiting from immunotherapy. 13 16

In recent years, multiple clinical scoring systems have been developed to guide antibody testing and, in some cases, empirical immunotherapy. Dubey et al. 24 introduced the Antibody Prevalence in Epilepsy (APE) score after analyzing clinical, magnetic resonance imaging (MRI), and CSF data from patients with new-onset or established epilepsy of unknown etiology. A score ≥ 4 showed high sensitivity (82.6%) and specificity (82.0%) for neuronal-antibody positivity. 24 To improve performance in patients with encephalopathy, memory loss, or neuropsychiatric symptoms, the same group 25 subsequently proposed the Antibody Prevalence in Epilepsy and Encephalopathy (APE2) score, in which a cutoff ≥ 4 yielded a sensitivity of 99% and a specificity of 93% for the presence of neuronal antibodies.

While the APE and APE2 scores share similarities, the APE2 score has an expanded maximum of 18 points (versus 15 for the APE), due to refined criteria such as separating faciobrachial dystonic seizures from facial dyskinesias and incorporating additional MRI findings. It also narrows the relevance of malignancies to those diagnosed within 5 years. Both scores are primarily suited to the identification of acute symptomatic seizures due to autoimmune encephalitis, rather than AAE. 17

In 2021, De Bruijn et al. 23 developed the Antibody Contributing to Focal Epilepsy Signs and Symptoms (ACES) score based on a multicenter cohort of patients with focal epilepsy of unknown etiology, excluding those with signs of encephalitis. The study included individuals with epilepsy duration between 2 and 18 years. A multivariate analysis identified six predictive variables: history of autoimmune disease, behavioral changes, cognitive symptoms, speech disturbance, autonomic symptoms, and mesial temporal-lobe hyperintensities on MRI. An ACES score ≥ 2 demonstrated 100% of sensitivity (95%CI = 81.4–100) and 84.9% of specificity (95%CI = 67.9–100). Although all antibody-positive patients were captured with this threshold, 14% of those meeting this criterion had negative antibody panels, reinforcing that the score is better suited for screening rather than diagnosis. Notably, nearly 70% of antibody-positive patients would have been missed using the APE2 score alone, underscoring the importance of tailored tools for chronic epilepsy scenarios. 23

Also in 2021, the Antibody Prevalence in Epilepsy before Surgery (APES) score was introduced 19 to identify candidates for antibody testing during preoperative evaluation in drug-resistant focal epilepsy of unknown etiology. This is especially relevant given the poorer postoperative outcomes observed in antibody-positive patients.

Adapted from the APE2 score, the APES score includes additional variables such as positron-emission tomography and computed tomography (PET-CT) abnormalities, focal neurological signs at seizure onset, and personal or family history of autoimmunity. Cerebrospinal fluid data were excluded, given that lumbar puncture is not routinely performed in preoperative protocols. A cutoff ≥ 4 showed improved sensitivity (100% versus 71.4%) with comparable specificity (44.3% versus 49.4%) relative to the APE2 score. Among individuals with complete datasets ( *n* = 60), the predictive accuracy remained consistent. 19 However, the score still requires external validation before routine clinical implementation.

Another scoring tool proposed in 2021 is the Antibody in Drug-Resistant Temporal-Lobe Epilepsy (ARTE) score, 26 aimed at identifying antibody-positive individuals among patients with pharmacoresistant TLE of unknown cause. The study assessed 27 adults with disease duration > 12 months. Clinical variables were compared between antibody-positive and -negative patients to derive the score. A cutoff ≥ 1 yielded 100% of sensitivity and 46.1% of specificity, while ≥ 3 increased the specificity to 100% but reduced the sensitivity to 35.7%. 26 The small sample size limits generalizability and highlights the need for validation in independent cohorts.

Table 2 outlines the criteria for each scoring system, whereas Table 3 provides a direct comparison of them.

**Table 2:** Proposed scores to predict autoimmune-associated epilepsy with autoantibodies positivity (antibody-associated epilepsy)

Score	Criteria	Pt	Score	Criteria	Pt
**APE**	New-onset, rapidly-progressive mental-status changes in 1–6 weeks, or new-onset seizure activity	1	**APE2**	New-onset, rapidly-progressive mental status-changes that developed over 1–6 weeks, or new onset seizure activity (within 1 year of evaluation)	1
	Neuropsychiatric changes	1		Neuropsychiatric changes	1
	Autonomic dysfunction	1		Autonomic dysfunction	1
	Viral prodrome, only to be scored in the absence of underlying malignancy	2		Viral prodrome to be scored in the absence of underlying systemic malignancy within 5 years of onset	2
	Facial dyskinesias or faciobrachial dystonic movements	2		Faciobrachial dystonic seizures	3
	Seizure refractory to at least 2 ASMs	2		Facial dyskinesias, to be scored in the absence of faciobrachial dystonic seizures	2
	CSF findings consistent with inflammation (CSF protein level > 50 mg/dL and/or lymphocytic pleocytosis > 5 cells/dL)	2		Seizure refractory to at least 2 ASMs	2
	Brain MRI showing signal changes consistent with limbic encephalitis	2		CSF findings consistent with inflammation (CSF protein > 50 mg/dL and/or lymphocytic pleocytosis > 5 cells/mcL)	2
	Presence of underlying malignancy			Brain MRI suggesting encephalitis	2
	TOTAL	15		Systemic cancer diagnosed within 5 years of neurological symptom onset	2
				TOTAL	18
**Score**	**Criteria**	**Pt**	**Score**	**Criteria**	**Pt**
**ACES**	Cognitive symptoms	1	**APES**	New-onset SE or flurry of seizures or progressive altered mental status (≤ 3 months)	1
	Behavioral changes	1		Triad: explosive symptom onset + unusual neuropsychiatric changes + MRI/PET changes	1
	Autonomic symptoms	1		Autonomic dysfunctions or autonomic seizures	1
	Speech problems	1		Viral prodrome, meningitis/encephalitis of unclear etiology	1
	Autoimmune diseases	1		Facial dyskinesias or faciobrachial dystonic seizures	2
	Temporal MRI hyperintensities	1		New focal CNS findings: speech changes, cerebellar signs, myoclonus, hyperekplexia, mutism, catatonia	1
	TOTAL	6		Refractory to at least 2 ASMs and/or refractory to antipsychotics	2
**Score**	**Criteria**	**Pt**		PET lobar hypo/hypermetabolism	1
**ARTE**	Cognitive symptoms	1		MRI: unilateral/bilateral mesial temporal sclerosis, inflammatory changes	2
	Autoimmune disease	1		Underlying malignancy	2
	Bitemporal independent interictal epileptiform discharges	1		Medical or family history autoimmunity	1
	History of SE	1		Systemic: weight loss ≥ 10 pounds, hyponatremia < 130 mmol/L, sleep disorder excluding OSA, neuromyotonia, peripheral-nerve hyperexcitability	1
	TOTAL	4		TOTAL	16

Abbreviations: ACES, Antibody Contributing to Focal Epilepsy Signs and Symptoms; APE, Antibody Prevalence in Epilepsy; APE2, Antibody Prevalence in Epilepsy and Encephalopathy; APES, Antibody Prevalence in Epilepsy before Surgery; ARTE, Antibody in Drug-Resistant Temporal-Lobe Epilepsy; ASM, antiseizure medication; CNS, central nervous system; CSF, cerebrospinal fluid; MRI, magnetic resonance imaging; OSA, obstructive sleep apnea; PET, positron-emission tomography; Pt, points; SE, status epilepticus.

**Table 3:** Comparison of the scores used to predict autoimmune-associated epilepsy with autoantibodies positivity (antibody-associated epilepsy)

Score	Population evaluated	Maximum score/Cut-off score	Commentaries
APE	Patients with new-onset or established epilepsy of unknown etiology	Maximum: 15 points; Cut-off: ≥ 4	Both scores are more useful for detecting acute symptomatic seizures secondary to autoimmune encephalitis rather than autoimmune-associated epilepsy
APE2	Validation of the APE score in patients with altered mental status, short-term memory loss, or neuropsychiatric dysfunction	Maximum: 18 points; Cut-off: ≥ 4	
ACES	Multicenter cohort of patients with focal epilepsy of unknown etiology but without symptoms suggestive of autoimmune encephalitis	Maximum: 6 points; Cut-off: ≥ 2	When comparing the APE2 and ACES, the latter performed better in chronic epilepsy settings, as nearly 70% of the patients would have been missed using the APE2 alone
APES	Adults with pharmacoresistant focal epilepsy of unknown etiology, without excluding those with mesial temporal sclerosis	Maximum: 16 points; Cut-off: ≥ 4	The APES was adapted from the APE2 by incorporating additional variables, such as PET-CT findings, focal neurological signs at onset, and personal or family history of autoimmunity
ARTE	27 adult patients with temporal-lobe epilepsy of unknown etiology and disease duration longer than 12 months	Maximum: 4 points; Cut-off: ≥ 2	The study included a small sample size and requires validation in independent cohorts

Abbreviations: ACES, Antibody Contributing to Focal Epilepsy Signs and Symptoms; APE, Antibody Prevalence in Epilepsy; APE2, Antibody Prevalence in Epilepsy and Encephalopathy; APES, Antibody Prevalence in Epilepsy before Surgery; ARTE, Antibody in Drug-Resistant Temporal-Lobe Epilepsy; PET-CT, positron-emission tomography and computed tomography.

In 2022, the ‘Obvious’ indications for Neural antibody testing in Epilepsy or Seizures (ONES checklist) was developed 27 through a literature review and subsequently evaluated for sensitivity and specificity in comparison with the APE2/ACES reflex score. This retrospective analysis included 96 patients who underwent neural antibody testing for epilepsy or seizures of unknown etiology. The APE2/ACES reflex score was defined as either an APE2 score ≥ 4, or an APE2 score ≤ 3 combined with an ACES score ≥ 2. The median interval from seizure onset to neural antibody testing was of 2 (range: 0–41) years, with 38% of the patients tested within 1 year of seizure onset. No significant difference in sensitivity or specificity was observed between the ONES checklist and the APE2/ACES reflex score. 27 However, as discussed regarding the APE and APE2 scores, the ONES checklist is more appropriate for acute presentations in which autoimmune encephalitis is suspected, and it may have limited applicability in chronic epilepsy without encephalitic features. Table 4 presents the ONES checklist, which adopts a distinct approach by emphasizing clinical features suggestive of neural antibody positivity that might otherwise go unrecognized by clinicians.

**Table 4:** The ONES Checklist

1) Are any of the following present?	Antibody/antibodies of most relevance
**1a) Perform panel-based neural antibody testing including anti-MOG if any of the following are present:**	
**Brain magnetic resonance imaging**	
Cortical T2-FLAIR hyperintense lesion(s) with or without involvement of the underlying white matter, in temporal relation to seizure onset	Anti-MOG, NMDAR, GABAAR, mGluR5
Large (> 1–2 cm) T2-FLAIR hyperintense lesion(s) involving the white matter suggestive of non-MS demyelination, in temporal relation to seizure onset	Anti-MOG, may overlap with anti-NMDAR
**Clinical**	
Optic neuropathy or myelopathy of unknown etiology, in temporal relation to seizure onset	Anti-MOG, may overlap with anti-NMDAR
**1b) Perform panel-based neural antibody testing including anti-GlyR if the following is present:**	
**Clinical**	
Prominent stiffness, spasms, rigidity, and/or hyperekplexia of unknown etiology, in temporal relation to seizure onset	Anti-GlyR, amphiphysin, DPPX, GAD65
**3) If all of the above features are absent, perform panel-based neural antibody testing excluding anti-MOG and anti-GlyR if any of the following are present:**	
**Brain magnetic resonance imaging**	
T2-FLAIR hyperintensity restricted to the medial temporal lobe(s) without atrophy, in temporal relation to seizure onset	Various
Linear radial perivascular enhancement, in temporal relation to seizure onset	Anti-GFAP may overlap with anti-NMDAR
**Biochemical**	
New (within 1 year), refractory temporal-lobe or presumed temporal-lobe seizures, with serum sodium < 130 mEq/L, of unknown etiology	Anti-LGI1
**Clinical/semiological/electroencephalographic**	
Distinguishable central or peripheral nervous system dysfunction of unknown etiology, in temporal relation to seizure onset	Various
Musicogenic seizures	Anti-GAD65
Faciobrachial dystonic seizures	Anti-LGI1
New (within 1 year), refractory temporal-lobe or presumed temporal-lobe seizures, with pilomotor seizures	Anti-LGI1
New (within 1 year), refractory temporal-lobe or presumed temporal-lobe seizures, with paroxysmal dizziness spells	Anti-LGI1
New (within 1 year), refractory, temporal-lobe or presumed temporal-lobe seizures, beginning after 50 years of age	Anti-LGI1, CASPR2
Refractory temporal-lobe or presumed temporal-lobe seizures, with anti-GAD65-associated systemic autoimmunity	Anti-GAD65
**Historical**	
New (within 1 year) seizures, beginning within 2 years of tumor diagnosis	Various
New (within 1 year) seizures, beginning within 1 year of the last immune checkpoint inhibitor treatment	Various
**2) If all of the above features are absent, perform panel-based neural antibody testing excluding anti-MOG and anti-GlyR if any of the following are present:**	
New (within 1 year) seizures, beginning or worsening within 3 months of the last antiviral treatment for *herpes simplex* -virus encephalitis	Anti- NMDAR

Abbreviations: CASPR2, contactin-associated protein-like 2; DPPX, dipeptidyl-peptidase- like protein 6; FLAIR, fluid-attenuated inversion recovery; GABAAR, γ-aminobutyric acid type-A receptor; GAD65, glutamic acid decarboxylase 65; GFAP, glial fibrillary acidic protein; GlyR, glycine receptor; LGI1, leucine-rich glioma-inactivated 1; mGluR5, metabotropic glutamate receptor 5; MOG, myelinoligodendrocyte glycoprotein; MS, multiple sclerosis; NMDAR, N-methyl-D-aspartate receptor; ONES, ‘Obvious’ Indications for Neural Antibody Testing in Epilepsy or Seizures.

**Figure 2:**
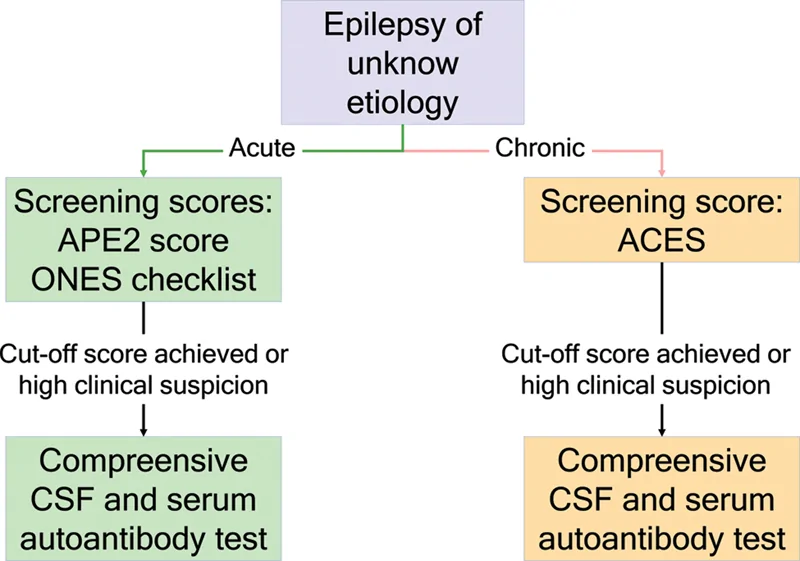
Abbreviations: APE2, Antibody Prevalence in Epilepsy and Encephalopathy; ACES, Antibody Contributing to focal Epilepsy Signs and Symptoms; CSF, cerebrospinal fluid; ONES, ‘Obvious’ Indications for Neural Antibody Testing in Epilepsy or Seizures. Selection of screening tools based on the clinical presentation of epilepsy.

Once appropriate candidates are identified, either through clinical assessment or validated screening tools (selected according to whether the presentation is acute or chronic, as illustrated in Figure 2 ), the next challenge is determining which antibodies to test for. Because the clinical phenotype rarely suggests a specific antibody, a broad, parallel testing approach is preferred to avoid delays and costs associated with sequential testing. 28 Simultaneous testing of the serum and CSF is also recommended, as certain antibodies may be compartment-specific; for instance, NMDAR antibodies may be negative in serum, while LGI1 antibodies may be absent in CSF. 10 28 29

False positives can occur, particularly in commercial panels, and they must be interpreted in the context of the clinical scenario. Indeed, in some cases, more than one neuronal autoantibody may be identified. 11 Conversely, some patients with classic autoimmune phenotypes may test negative for all known antibodies, reinforcing the need for diagnostic criteria that integrate clinical and laboratory features, as already occurs in rheumatology. 17

The testing methodology depends on the antibody type. Antibodies against intracellular antigens can be detected using Western blot, line assay, enzyme-linked immunosorbent assay (ELISA), fixed tissue-based assay (TBA), cell-based assay (CBA), or radioimmunoassay (RIA). Antibodies targeting surface antigens are typically assessed using immunohistochemistry, RIA, non-fixed or post-fixed TBA, and CBA—the latter being the most widely-adopted method. Non-fixed or post-fixed TBA involves rodent brain-tissue incubation followed by microscopy or immunofluorescence, and it is used for antibody discovery and CBA screening. 11 12 15 30

For anti-GAD65 antibodies specifically, commercial tests include ELISA, RIA, TBA, and line immunoassays. The ELISA and RIA are highly-sensitive, but they may detect low-titer antibodies that lack clinical significance; only high-titer anti-GAD65 antibodies are considered relevant in autoimmune neurological syndromes. 12 Testing the serum and CSF is advisable, as some patients show intrathecal synthesis despite low or undetectable serum levels. 31

Confirmatory testing using multiple techniques, such as combining CBA and immunofluorescence, enhances diagnostic accuracy, especially since different CBA methods (live versus fixed cells) can vary in sensitivity and specificity. 17 19 For intracellular antibodies, the preferred strategy includes fixed TBA and line assay, with the latter often showing higher sensitivity compared with indirect immunohistochemistry. 12 In Brazil, for example, the 2024 Consensus on Autoimmune Encephalitis 32 recommends a combined approach using CBA and TBA, along with paired serum and CSF analyses, for patients meeting the criteria for possible autoimmune encephalitis. Therefore, antibody interpretation must integrate clinical phenotype, disease chronology, antibody titer, and compartment (serum versus CSF), rather than relying solely on seropositivity.

## ADDITIONAL TESTS IN THE DIAGNOSTIC WORKUP

In AAE patients, EEG remains a useful tool to evaluate encephalopathy, epileptiform discharges, and seizure activity. However, there are no EEG patterns specific enough to reliably identify AAE outside the context of autoimmune encephalitis. 33 The ‘extreme delta brush’ pattern, for example, is considered characteristic of anti-NMDAR encephalitis, but it is not typically observed in other antibody-associated epilepsies.

On the other hand, VEEG monitoring is particularly informative. Subtle clinical manifestations may go unnoticed by patients, resulting in an underestimation of seizure frequency; VEEG provides a more accurate assessment of seizure burden, and it is also valuable for monitoring treatment response to immunotherapy and ASMs. 1 22

Brain MRI remains a cornerstone in the evaluation of epilepsy, supported by well-established guidelines by the ILAE. Repeat imaging using the harmonized neuroimaging of epilepsy structural sequences (HARNESS)-MRI protocol—which includes high-resolution three-dimensional (3D) T1-weighted, 3D fluid-attenuated inversion recovery (FLAIR), and two-dimensional (2D) coronal T2-weighted sequences—is recommended in cases in which previous MRI is unavailable or suboptimal. Importantly, neuroimaging findings should be interpreted in conjunction with electroclinical data, especially in the context of unexplained seizure worsening, rapid cognitive decline, or new-onset neuropsychiatric symptoms. 34

Positron emission tomography and computed tomography with 18F-fluorodeoxyglucose (18F-FDG), which assesses regional cerebral glucose metabolism, can offer additional diagnostic information. This technique is widely used in the evaluation of brain tumors and inflammatory disorders. 35 In AAE, most available data focus on cases of autoimmune encephalitis or acute symptomatic seizures. For the early detection of inflammatory brain involvement, PET-CT has been proposed as a modality more sensitive than MRI. 35 36

Inflammatory markers such as C-reactive protein (CRP) and the erythrocyte sedimentation rate (ESR) may be elevated in infectious or systemic inflammatory states. While nonspecific, some diagnostic protocols 37 include these markers as part of the workup, particularly to screen for systemic autoimmune or inflammatory conditions.

Several systemic autoantibodies have also been investigated in epilepsy. In a 2015 observational study 38 involving 319 patients without known autoimmune disease, the presence of antinuclear antibodies (ANA), anti-β2-glycoprotein 1, anticardiolipin IgG, antimicrosomal antibodies, and antithyroglobulin was evaluated. At least one autoantibody was detected in 23.5% of the participants, with the following prevalences: anticardiolipin (9.4%), antimicrosomal (7.5%), ANA (5.6%), anti-β2-glycoprotein (5.6%), and antithyroglobulin (3.25%).

Regarding the role of anti-thyroid peroxidase (anti-TPO) antibodies, a study 39 comparing the prevalence of antithyroid antibodies in 42 patients with adult-onset TLE, stratified by known and unknown etiology, found a higher frequency of anti-TPO positivity among patients with unknown etiology. Furthermore, late-onset TLE associated with antithyroid antibodies tended to occur in middle-aged women and was frequently accompanied by cognitive decline and other autoimmune disorders, including anti-GAD65 positivity. 39 However, anti-TPO antibodies are relatively common in the general population (particularly among women), and their presence may represent an epiphenomenon rather than a direct pathogenic mechanism. Thus, although these findings contribute to the ongoing debate regarding the controversial role of anti-TPO antibodies in steroid-responsive encephalopathy associated with autoimmune thyroiditis (SREAT), anti-TPO positivity alone should not be considered evidence of AAE. These data reinforce the importance of a comprehensive and clinically-contextualized autoimmune evaluation in selected patients.

Complement-system analysis has emerged for the identification neuroinflammation. Activation of the complement within the CNS is increasingly recognized as a contributor to tissue injury in neuroinflammatory and neurodegenerative diseases. In animal and human studies, dysregulation of the classical complement pathway has been associated with poor seizure control. A study 40 comparing 157 patients with epilepsy to 54 healthy controls, for instance, found significantly-elevated plasma complement component 4 (C4) levels among individuals with drug-resistant epilepsy, suggesting a role for complement activation in pharmacoresistance.

In epilepsy, CSF analysis is typically nonspecific, but it can offer valuable insights in certain contexts. Pleocytosis is rare following seizures, although it may occur when lumbar puncture is performed within hours to days after a seizure. When present, further testing should be performed to exclude infectious or immune etiologies such as meningitis or encephalitis. In contrast, elevated CSF protein levels are more frequently observed—reported in 10 to 61% of the patients, depending on the series—and they are believed to reflect blood-brain barrier dysfunction rather than a specific etiologic factor. 29 Establishing a clear protein cutoff to distinguish inflammatory from postictal changes is difficult due to variability in methodology and inconsistent exclusion of confounding conditions across studies.

Oligoclonal bands (OCBs) are rare in epilepsy patients (reported in 0–10% of the patients), but, when present, they may suggest an underlying autoimmune mechanism. These bands are most frequently observed in cases of anti-NMDAR, anti-GAD65, and anti-GABAB receptor encephalitis. Some authors 29 advocate that the presence of an *inflammatory CSF profile* —defined by pleocytosis and OCB positivity—should prompt investigation for immune epilepsy, even in the absence of detectable neural autoantibodies.

More recently, research has focused on cytokine profiling as a potential biomarker strategy. A study comparing patients with drug-resistant epilepsy and positive neuronal autoantibodies to those without antibodies found significant differences in CSF levels of interleukin-13 (IL-13), RANTES (also known as chemokine ligand-5, CCL5), and vascular endothelial growth fator (VEGF). Although promising, these findings require replication in larger, prospective cohorts before being incorporated into the routine clinical practice.

## TREATMENT AND THERAPEUTICAL TRIALS

A 2018 systematic review 42 demonstrated that the efficacy of ASMs in AAE is limited. Only ∼ 10% of the patients achieved seizure freedom with ASM monotherapy, and fewer than 15% experienced a reduction greater than 50% in seizure frequency. These findings underscore the need to consider adjunctive immunotherapy in selected patients. 30

Regarding immunotherapy, a case series 43 evaluated 81 patients without clinical features suggestive of limbic encephalitis (although a subgroup presented with psychiatric symptoms, movement disorders, cognitive impairment, or coexisting autoimmune disease), with a mean disease duration exceeding 6 years. Among antibody-positive patients who received immunotherapy, 75% (9/12) achieved a reduction greater than 50% in seizure frequency, and 50% became seizure-free.

In 2016, Bakpa et al. 15 published a review including 34 studies (involving 1,451 patients), reporting clinical improvement in 81% of the patients following immunotherapy, with 67% achieving seizure control within 10 months. However, most included studies involved patients with associated encephalopathy, suggesting that cases of autoimmune encephalitis may have been incorporated, thereby limiting the precision and generalizability of these estimates to isolated AAE.

Diagnostic delay is another factor influencing treatment response. In a cohort of 86 patients with drug-resistant focal epilepsy of unknown etiology, 19 the mean interval between seizure onset and comprehensive presurgical evaluation, including assessment for immune-mediated causes, was of 14.2 years. Such delay may contribute to reduced responsiveness to immunotherapy due to the development of irreversible structural or network alterations.

More invasive and costly interventions typically reserved for drug-resistant epilepsy, such as epilepsy surgery and neurostimulation devices, appear to be less effective in patients with immune-mediated etiologies. A retrospective multicenter study 44 demonstrated that seizure outcomes following temporal-lobe surgery in patients with neuronal antibodies were inferior to those observed in other clinical contexts.

Given the limited efficacy of the usual therapeutic approaches, once an immune etiology is reasonably established, a trial of immunotherapy should be considered. However, treatment response depends on the timing of the intervention and the type of autoantibody involved. In cases associated with intracellular antibodies, such as anti-GAD65, complete seizure resolution is uncommon. 10

Importantly, the evidence supporting immunotherapy in chronic AAE remains largely derived from observational cohorts and retrospective series, frequently including patients with encephalitic features. Randomized controlled trials are lacking, and response rates may reflect early treatment of active inflammation rather than reversal of established epileptogenic networks. Once structural or network remodeling becomes consolidated, seizure persistence may no longer represent ongoing immune activity.

In general, during the induction phase, the first-line therapies include intravenous methylprednisolone (IVMP), intravenous immunoglobulin (IVIg), and plasma exchange (PLEX). The second-line therapies typically include rituximab and cyclophosphamide. In the maintenance phase, the options include gradual tapering of first-line agents, continuation of second-line therapy, or the addition of an alternative immunotherapy, such as mycophenolate mofetil or azathioprine. 45

Empirical immunotherapy should be reserved for patients with high pretest probability of immune etiology and objective supportive features (such as inflammatory CSF profile, high-titer neuronal antibodies, compatible MRI findings). A time-limited therapeutic trial with predefined response criteria may help balance potential benefits against the risks of prolonged immunosuppression.

## CHALLENGES AND FUTURE DIRECTIONS

Epilepsy care worldwide is characterized by significant disparities, particularly in low- and middle-income regions such as Latin America, where access to essential diagnostic tools—including EEG, MRI, and ASMs—remains limited. 46 To guide the rational adoption of new diagnostic technologies, Schaafsma et al. 47 proposed a hierarchical framework that evaluates test characteristics (such as sensitivity and specificity), diagnostic yield (that is, improvement in the accurate classification of disease versus non-disease), clinical impact, and cost-effectiveness. When assessed using this framework, autoantibody testing in selected patients with epilepsy demonstrates meaningful clinical usefulness.

Autoantibody testing is fundamental for the diagnosis of autoimmune conditions, including AAE. Early identification of an immune-mediated etiology may enable the timely initiation of immunotherapy and potentially mitigate cognitive decline and contribute to improved long-term outcomes, especially in patients undergoing a preoperative evaluation. Nonetheless, financial and systemic barriers frequently limit the availability of antibody testing in resource-constrained settings. These include high test costs, insufficient laboratory infrastructure, and limited coverage by public or private health systems.

To address these barriers, it is imperative to foster the development of cost-effective testing platforms, invest in local laboratory capacity, encourage public–private collaborations, and multicentric consortia. Such strategies could enable earlier and more accurate diagnoses, ultimately improving care for patients with suspected AAE across diverse healthcare systems. Recognizing and addressing these diagnostic and structural challenges will be critical to advance equitable, evidence-based care for epilepsy patients with potential autoimmune etiologies.

Autoimmune-associated epilepsy remains a diagnostically-challenging entity at the intersection between epileptology and neuroimmunology. A structured, probability-based approach integrating clinical phenotype, validated screening tools, using comprehensive autoantibodies panels in CSF and serum tested by appropriate techniques, and contextual antibody interpretation is essential to avoid underdiagnosis and overtreatment.
